# Supplementary material for: Conditional chemoconnectomics (cCCTomics) as a strategy for efficient and conditional targeting of chemical transmission
Source: eLife. 2024 Apr 30;12:RP91927. doi: 10.7554/eLife.91927 (PMC11060718; doi:10.7554/eLife.91927)
Supplement: Figure 3—source data 3. [file elife-91927-fig3-data3.zip › Figure 3 source data3 part 1.zip!/Figure 3-Source data 3-part1/1067-9-CRISPResso_Report_c33kf3u3.zip!/CRISPResso_on_1067_10/2a.Nucleotide_percentage_quilt.pdf]

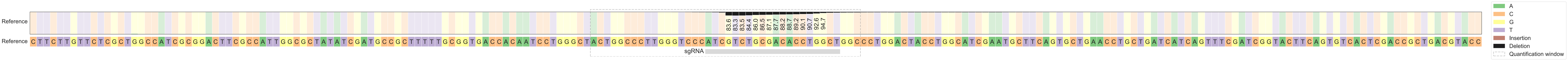
Reference
Reference
83.6
83.3
83.5
84.4
86.0
86.5
87.1
87.7
88.2
88.7
89.2
90.1
90.7
92.6
94.7
sgRNA
A
C
G
T
Insertion
Deletion
Quantification window
